# Linking employee work-related personal resources and perceived employability: the mediating role of work engagement

**DOI:** 10.3389/fpsyg.2026.1814522

**Published:** 2026-04-01

**Authors:** Dalia Bagdžiūnienė, Laima Okunevičiūtė Neverauskienė

**Affiliations:** 1 Institute of Psychology, Vilnius University, Vilnius, Lithuania; 2 Lithuanian Centre for Social Sciences, Vilnius, Lithuania

**Keywords:** personal resources, work engagement, perceived employability, mediation, employees

## Abstract

**Introduction:**

In the modern dynamic labor market, perceived employability, defined as an individual’s self-assessed capability to obtain and retain employment and to make labor-market transitions, becomes particularly important. In this context, it is relevant to empirically disclose the role of work-related personal resources and the mechanism linking them to perceived employability in the external labor market.

**The aim:**

Building on the Job Demands-Resources framework, this study examines the relationships between work-related personal resources and perceived external employability and the mediating role of work engagement.

**Methods:**

The study sample included 1,032 employees from various Lithuanian organizations: 54.8 percent were female, and the mean age was 42.5 years (*SD* = 11.85). The survey questionnaire included self-report measures assessing personal resources (adaptability, job crafting, and strength use), work engagement, and perceived employability.

**Results and conclusion:**

Structural equation modeling reveals that adaptability and job crafting were positively related to work engagement and perceived employability; work engagement mediated the relationships among adaptability, job crafting, and the utilization of strengths and perceived employability, highlighting the motivational pathway proposed by the Job Demands–Resources framework.

**Implications:**

The results extend the theory by showing that employability is a key outcome of employees’ investment in personal resources and engagement. In practice, the findings suggest that organizations and employees themselves can enhance perceptions of employability by developing a range of personal resources, such as adaptability, job crafting, and strengths utilization, and by promoting work engagement. The study provides empirical evidence supporting the role of work engagement in linking personal resources to perceived employability in today’s labor markets.

## 1 Introduction

The world of work is an inexhaustible engine of social progress, a source of innovation, and a space for research relevant to society and its members. Employability is one of those phenomena that encompasses and combines the interests of society, organizations, and individuals, helping to assess employment processes, assumptions, and consequences, ranging from labor market dynamics to issues of professional career development for each individual. As Vanhercke et al. (2014) state, the broadly defined term employability combines the words “employment” and “ability” and describes a person’s ability to find and keep a job.

Processes in the labor market, employment, and employability can be examined from various perspectives, broadly divided into macro-, meso-, and micro-levels (Vanhercke et al., 2014). The macro-level approach focuses on society, employment policy, and the labor market. The meso-level concerns personnel policy, recruitment, retention, career management, professional growth strategies, and measures implemented within organizations. The micro-level is the individual-level approach to employability, focusing on factors that ensure opportunities for employees, graduates, or unemployed individuals to get employed, retain employment, or move to another organization. Our research was conducted from the individual-level perspective. Each of these levels is intertwined with various challenges and opportunities to consider when making decisions at the state policy level, planning and implementing organizational measures, or managing the individual professional career.

From 2020 to 2024, Lithuania was in a relatively strong position in the labor market compared with other EU countries. During this period, the employment rate in Lithuania rose by 2.5 percentage points, while the EU average rose by 4.1 percentage points. In 2024, the average annual total employment rate in Lithuania was 79.2 percent, while in the European Union it was 75.8 percent (Eurostat, 2025). The unemployment rate in Lithuania has also been rising in recent years. From 2022 to 2024, it increased from 5.9 to 7.1 percent. In 2024, the average annual unemployment rate was 7.1 percent, exceeding the EU average of 6.0 percent (Eurostat, 2025). In the context of our research, it is important to note that the number of job vacancies was also increasing. According to data from the State Data Agency (Valstybės duomenų agentūra, 2025), job vacancies in Lithuania steadily increased from 2020 to 2024, reaching 28.2 thousand in 2024. As Bloom et al. (2018) indicate, “between 2010 and 2030, global labor markets will face the daunting task of generating roughly three-quarters of a billion new jobs” (p. 2).

Vacancies, in themselves, do not guarantee that there will be people willing and able to fill them. Various factors influence the labor-market activity of working-age employed, unemployed or graduated individuals, including their perceptions of personal employability. Berntson et al. (2006) were among the first to point out that employment depends not only on actual professional abilities to find a job but also on self-assessment of employability opportunities, which was described as “the individual’s perception of his or her possibility to achieve a new job” (p. 225).

Perceived employability of employees can be viewed in different ways, considering factors such as age, gender, occupation, and even place of residence. Studies indicate that individuals with higher education, younger age, and more work experience generally rate their personal employability higher (De Lange et al., 2021; Bennett et al., 2022). Research confirms that the work performed is also important for enhancing a person’s chances of employment in another organization by providing professional and social experience, helping to acquire, highlight, and strengthen professional skills, and creating social connections necessary for employment (van Hetty Emmerik et al., 2012). Research on work-related personal employability resources supplements this field by emphasizing individual factors that not only enable effective performance but may also improve workers’ perceptions of external employability opportunities.

In this study, we focus on employees from various Lithuanian organizations. The identification of determinants of perceived employability is facilitated by applying the Job Demands-Resources model (Bakker and Demerouti, 2017; Bakker et al., 2023). According to Berntson et al. (2006), research on the antecedents of perceived employability should examine a broad range of individual factors. We conceptualize employee adaptability, job crafting, and strengths use as work-related personal resources that reflect the capacity to adapt to job requirements, proactively shape the work environment, and mobilize personal assets to meet job demands. These resources may foster work engagement, motivate achievement of work goals, accumulate professional experience, and strengthen subjectively evaluated personal employability.

The overall aim of our study is to investigate the relationships between employees’ work-related personal resources and perceived employability and to examine the mediating role of work engagement in these relationships. We pursue this goal in three steps. First, we test the expectation that employees who place greater value on personal resources will report higher perceived employability. Second, we expect that those more engaged in their work will also report higher perceived employability. Third, we expect that work engagement will mediate the relationships between personal resources and perceived employability, thereby explaining the mechanism underlying these relationships.

## 2 Theoretical framework

### 2.1 Perceived employability

In its general sense, the term „employability “refers to “an individual’s chance of a job in the internal and/or external labor market” (Forrier and Sels, 2003, p. 106). Employability has been widely studied among the unemployed (Fernández-Valera et al., 2020), students (Baluku et al., 2021), and graduates beginning their professional careers (Grosemans et al., 2023). In recent years, attention has increasingly focused on the employability of working individuals. As a personal resource, employability has been shown to positively influence boundaryless career orientations (Redondo et al., 2024), support career continuity (Lo Presti and Pluviano, 2016; Seibert et al., 2024), and strengthen active job search behavior (De Battisti et al., 2016; Biramo et al., 2025). Due to economic, technological, and structural changes, a person’s career is no longer tied to a single organization. Career continuity is more assured by an individual‘s professional potential and the demand for their competencies in the labor market than by an employer’s guaranteed career opportunities within a single organization (Peeters et al., 2020). As Forrier and Sels (2003) state, a successful career is believed to be assured by having the appropriate personal capacities to remain continuously employable in the internal and external labor markets throughout one’s working life.

The agentic perspective in employability research emphasizes that individuals are responsible for managing their careers and that career success, employment, and retention depend more on them than on their employers (Forrier et al., 2018). According to three main assumptions of the employability studies based on the agentic perspective, employability is a personal worth, employable individuals themselves control their careers, are free to make career-related decisions, and, lastly, the outcomes of employability first of all have a positive value for employees, as it encourages professional development, personal growth, and well-being (Vanhercke et al., 2016) and increases job search intensity and reemployment in case of job loss (Koen et al., 2013). In this context, the importance of the employee’s own view of personal employability, i.e., perceived employability, increases.

Perceived employability reflects an individual’s self-assessed ability to obtain and retain employment and to make labor-market transitions (Vanhercke et al., 2014) or one’s ability to realize job opportunities within and between employers over time” (Fugate et al., 2021, p. 266). De Cuyper and De Witte (2010) highlighted perceived employability as a guarantee of job security in a changing professional environment. Employment opportunities can be perceived within the current organization, for example, when an individual seeks career advancement (internal employability), or when considering the possibility of working with another employer (external employability) (Vanhercke et al., 2014; Van Harten et al., 2022; Forrier et al., 2015). In this study, we examine employee-perceived employability in the external labor market, which refers to one’s employment opportunities independent of the organization in which the individual works.

From the perspective of the Job Demands–Resources model (Bakker and Demerouti, 2017; Bakker et al., 2023), we examine adaptability, job crafting, and strengths use behaviors as work–related personal resources and valued characteristics that contribute to optimal employee functioning, control over job processes and the work environment, and may be significant to employees’ perceptions of employability in the labor market.

### 2.2 The job demands-resources model

The Job Demands–Resources model (Bakker and Demerouti, 2017; Bakker et al., 2023) integrates traditions in research on work-related stress and motivational processes and is used to assess the significance of demands, resources, and psychological states (burnout and work engagement) for employee job performance (Lazauskaite-Zabielske et al., 2018), organizational commitment (Akkermans et al., 2019), well-being (Vanhercke et al., 2016), work-life balance (Brough et al., 2020), and other outcomes. We analyse perceived employability as one of these consequences, linking the current job to the continuation of a professional career with the current employer or to a move to another organization.

Job demands—the physical, psychological, social, or organizational aspects of work that require sustained physical and/or psychological (cognitive and emotional) effort and are associated with physiological and/or psychological costs (Bakker and Demerouti, 2017; Bakker et al., 2023). High work demands, long working hours, time pressure, and work overload require significant time, energy, and personal effort, deplete internal employee resources, and are directly associated with stress and burnout processes (Bakker et al., 2023). On the other hand, the requirements for a specific position define the content of the work, for which a worker not only uses (adapts) professional competencies but also, while overcoming work challenges, has the opportunity to strengthen professional potential, learn, acquire new knowledge and skills, and improve in the profession (Fajaryati et al., 2020).

Job resources—organizational, social, physical, or psychological job and personal characteristics that help achieve work goals and purposefully promote personal growth and development (Bakker and Demerouti, 2017). Resources provide the conditions necessary to meet work requirements; they are associated with motivation processes and many positive consequences for the organization and the employee (Schaufeli and Taris, 2013; Sánchez-Cardona et al., 2023; Galanakis and Tsitouri, 2022). When examining the relationships between job resources and employability, social support, feedback, autonomy, and job variety were found to positively impact employability (Jabeen et al., 2022; van Leeuwen et al., 2022; van Hetty Emmerik et al., 2012; Marzec et al., 2021).

Personal resources are aspects of the self that reflect individuals’ sense of their ability to control and influence their environment successfully (Hobfoll et al., 2003). In the JD-R model, personal resources are defined as positive self-evaluations, psychological characteristics, or traits (Schaufeli and Taris, 2013). These resources include self-efficacy, organization-based self-esteem, psychological capital, resilience, and personality traits. Like job-related resources, these characteristics can be examined as direct predictors of positive employee outcomes, including job satisfaction, well-being, and organizational commitment, or as predictors of intermediate motivational states, such as work engagement. Xanthopoulou et al. (2007) were among the first to argue that personal resources should be included in the Job Demands-Resources model. They found that self-efficacy, occupational-based self-esteem, and optimism positively influenced work engagement, mediated the relationship between job resources and engagement, and, together with job demands and job resources, explained variance in work engagement. Low et al. (2020) found that personal resources (career-enhancing strategies and personal initiative) predicted perceived employability under both normal and harsh labor conditions.

As noted earlier, perceived employability can also be viewed as a resource that enables active participation in the labor market, whether seeking a job or maintaining employment in the current organization, and that supports career stability (Forrier and Sels, 2003; Redondo et al., 2024; Lo Presti and Pluviano, 2016; Seibert et al., 2024). Consistent with the Conservation of Resources theory (Hobfoll, 2002; Hobfoll et al., 2018), this resource needs to be created, protected, nurtured, and accumulated. In this way, work-related personal resources may enhance perceived employability, and, drawing on the Job Demands-Resources model, work engagement may explain the mechanism underlying these associations.

In this study, personal resources include adaptability, job crafting, and the use of strengths. The harmony of these employability assumptions is supported by Griffin et al. (2007) Performance theory, which holds that work processes in any position are dynamic and can change when new requirements are placed on employees or when the work environment or the functions performed change. Therefore, to act effectively, it is important to adapt to and accept these changes (adaptability), proactively model the work performance process to conserve external and personal resources, and know and apply one’s professional strengths (strengths use). All this helps to work creatively and may strengthen work engagement, a central factor in the motivational process within the Job Demands-Resources model and the outcome—perceived employability.

### 2.3 Personal resources, engagement, and perceived employability

#### 2.3.1 Adaptability and perceived employability

Employee adaptability is an individual’s ability to adapt to changing job demands, roles, and environments (Griffin et al., 2007). Pulakos et al. (2000) analyze adaptive employee performance as a multidimensional construct, with specific knowledge, skills, and abilities underlying dimensions such as handling work stress, solving problems creatively, etc. Adaptable workers tend to show openness to change, initiative, and resilience in the face of shifting work requirements or new, unfamiliar tasks that must be quickly mastered and performed efficiently. Taormina (2015) analyzed adaptability as one of four dimensions of the adult resilience construct and defined it as “. the capacity to be flexible and resourceful, and to cope with adverse environments and adjust oneself to fit into changing conditions” (p. 37). In that study, adaptability was not examined in relation to perceived employability; however, the author reported empirical data showing that employed professionals’ adaptability was significantly higher than that of unemployed respondents.

Forrier et al. (2009) identified adaptability as one of four dimensions of movement capital, directly linked to career mobility and, as research has shown, positively affecting perceived employability. The authors conducted a two-wave longitudinal study of working Flemish individuals and found a significant positive relationship between adaptability and perceived external employability (Forrier et al., 2015). In another study in line with the career mobility model, Peeters et al. (2020) conducted a longitudinal study examining the relationship between adaptability and perceived employability in employee samples from Belgian private- and public-sector organizations. The authors state that the central aspect of adaptability is openness to changes at work and the ability to adjust behavior in response to them. Adaptability was hypothesized to be related to perceived employability. However, the results of this study did not confirm a positive relationship between adaptability (openness to change) and perceived employability; instead, a negative relationship was observed in a private sector sample.

Building on the Job Demands-Resources model, we analyze workers’ adaptability as a personal resource and propose that it may enhance perceptions of employability, as individuals who can and are motivated to respond flexibly to changing work requirements may perceive themselves as better able to secure alternative employment opportunities outside the current organization. Therefore, employee adaptability may be expected to be positively related to perceived employability.

*H1*: Employee adaptability is positively related to perceived employability.

#### 2.3.2 Job crafting and perceived employability

Job crafting is broadly defined as an employee’s proactive response to job design aimed at improving performance and the work environment. A role-based approach, introduced by Wrzesniewski and Dutton (2001), links job crafting to changes in job design and the social environment. From this perspective, job crafting is defined as “the physical and cognitive changes individuals make in the task or relational boundaries of their work” (p. 179). Tims and Bakker (2010) developed the resource-based approach and incorporated job crafting into the Job Demands–Resources model as a process in which employees change the levels of job demands and job resources to “align them with their own abilities and preferences” (p. 4). The broader conceptualization presented by Vanbelle et al. (2023) emphasizes that it is appropriate to integrate these perspectives and analyze job crafting as a purposeful, proactive behavior that considers “both what employees may craft and why they engage in job crafting” (p. 6).

Job crafting, a proactive, change-oriented behavior, involves employees shaping their work environment to enhance work performance, engagement, and job satisfaction (Demerouti, 2026). It creates opportunities to apply professional knowledge and skills, to acquire new professional competencies, and supports professional growth. As Vanbelle et al. (2023) note, employees may adjust job demands and resources to align with their abilities and preferences. Applying job-crafting strategies makes work more enjoyable and enriched (Tims and Bakker, 2010; Tims et al., 2012), providing opportunities to increase work variety and perform more complex tasks. By crafting a job, employees can take on more responsibilities and apply innovations that require new skills and knowledge.

Employees may be more likely to improve their jobs (adapt to challenging job requirements and maintain a balance between job demands and resources) when they believe doing so will advance their careers. In other words, by learning and performing more complex tasks, employees build the professional foundation necessary for sustainable employability or promotion (Sartori et al., 2023). Akkermans and Tims (2017) show that expanding job resources or demands triggers personal growth and is positively associated with perceived employability. The effort invested in learning new skills to keep up with workplace changes helps maintain high employability. In this way, job crafting helps employees redesign their jobs to achieve personally valued outcomes at work (Wrzesniewski and Dutton, 2001; Tims and Bakker, 2010) and to enhance employability in the labor market (Irfan and Qadeer, 2020; Signore et al., 2023; Tims et al., 2012).

Job crafting involves proactive efforts to shape and redesign tasks, relationships, and cognitive perceptions of work (Wrzesniewski and Dutton, 2001) and to adjust job demands and resources to better align with employee strengths, interests, abilities, preferences, and optimal functioning at work (Tims et al., 2015). Crafting a job creates a more meaningful, motivating, and diverse work environment, applies a wider range of knowledge and skills, and strengthens professional competencies. This experience may increase confidence in professional capabilities and positively influence perceptions of employability outside the current organization. Therefore, we hypothesize that job crafting is positively related to perceived external employability.

*H2*: Job crafting is positively related to perceived employability.

#### 2.3.3 Strengths use and perceived employability

Strengths use behavior refers to the extent to which employees actively apply personal and professional strengths in performing work roles (Wood et al., 2011). Professional strengths, such as positive, well-developed skills, knowledge, and character traits, contribute to effective job performance and the ability to deliver consistent, high-level performance in a given activity (Clifton and Harter, 2003). Assessing the professional characteristics of candidates for a job, even at the stage of employee selection, helps define the profile of required qualities for a specific job position and justify employment decisions. However, it is only after starting work that the correspondence between professional competencies and the position’s requirements becomes apparent. Therefore, when discussing an employee’s level of professionalism, the most important consideration is not how many qualities he has or what they are, but whether and how he uses them, and what features of the work and social environments create favorable conditions for applying his strengths (Van Woerkom et al., 2016).

As Forrier et al. (2015) argued, the concept of employability centers on personal strengths that enable participation in the labor market and the maintenance of employment. The links between employability and an individual’s knowledge, skills, and abilities are well grounded in theory (Fugate and Kinicki, 2008; Vanhercke et al., 2014) and supported empirically (Forrier et al., 2015; Peeters et al., 2020). In the context of our study, research examining the links between another aspect of strengths - strengths use - and perceived employability is relevant; however, these links have been examined in only a few studies. For example, Gürbüz et al. (2022a) found that strengths use was positively related to sustainable employability and mediated the relationships between inclusive leadership and human resource practices and sustainable employability. In another study, Matsuo (2022) found that strengths use behavior was positively related to perceived employability and mediated the relationship between supervisor support for strengths use and perceived employability. Having competencies is not the same as using them; only using them helps active engagement in work, achieving work goals, revealing areas of professional development, fostering mastery experiences, and may also increase the evaluation of one’s abilities for employment. Thus, we hypothesize that using strengths at work may be positively associated with employee perceptions of external employability.

*H3*: Strengths use is positively related to perceived employability.

#### 2.3.4 Work engagement and perceived employability

Work engagement is “a positive, fulfilling, work-related state of mind that is characterized by vigour, dedication, and absorption” (Schaufeli et al., 2002, p. 74). Engagement indicates whether the work is stimulating, whether employees are willing to devote time and effort to it (the vigour, energy component), whether it is significant and meaningful (the dedication component), and whether the activity is attractive and encourages full concentration (the absorption component). Engaged employees are energetic, work independently, enjoy their work, and describe post-work fatigue as a pleasant state because it is associated with positive achievements and satisfaction with results (Bakker and Demerouti, 2017). Separate studies have found positive links between work engagement and job performance (Corbeanu and Iliescu, 2023), innovative behavior (Ali et al., 2022), and citizenship behavior (Gupta et al., 2017). Mazzetti et al. (2023), in a recent meta-analytic study based on 113 independent samples from prior research, reported that work engagement positively correlated with job satisfaction, job commitment, performance, and health, and negatively correlated with turnover intention and psychological distress.

When it comes to research on work engagement and perceived employability, there are only a few studies worth mentioning. Gürbüz et al. (2023) found that work engagement mediated the relationship between sustainable employability (an independent variable) and two dependent variables, task performance and job satisfaction. Liu et al. (2022) surveyed nurses during the COVID-19 pandemic and found that employability, as a boundary condition, negatively moderated the relationship between employee health and work engagement. Zhang and Liu (2023) found that employability, as a contextual variable, negatively moderated the relationship between workplace relational civility and nurses’ engagement.

In this study, we analyze work engagement as a prerequisite for perceived employability within the Job Demands-Resources model. Work that gives employees a sense of meaning, energizes them, and immerses them helps reveal one’s professional potential. Work engagement is an active state; it is expressed in work that draws on knowledge, skills, and abilities. Engaged workers accumulate experience by applying professional knowledge and skills, learn, and acquiring new competencies (Gürbüz et al., 2023). The results and experience achieved lay a foundation for professionalism, contribute to achieving career goals, and strengthen confidence in employability opportunities in the labor market (Matulcikova and Brevenikova, 2015). Thus, we expected that perceived employability, defined as employees’ perceptions of their ability to obtain and maintain employment (Vanhercke et al., 2014), may result from work engagement.

*H4*: Work engagement is positively related to perceived employability.

#### 2.3.5 The mediating role of work engagement

Based on the above discussions, empirical evidence and the theoretical background were presented to propose the direct effects of personal resources (adaptability, job crafting, and strengths use) on perceived employability. Along the motivational path of the Job Demands-Resources model, personal resources may be indirectly linked to perceived employability through work engagement as a mediating variable. As Bakker (2022) states, “work engagement refers to a motivational and fulfilling state characterized by high levels of mental and physical energy, enthusiasm about and dedication to work, and complete absorption in work activities” (p. 37). Work engagement can be stimulated by various organizational, social, work, and personal resources (Galanakis and Tsitouri, 2022) and leads to a wide range of outcomes for organizational, team, and individual performance (Corbeanu and Iliescu, 2023). Research also shows that work engagement serves as a mediator. For example, Gupta et al. (2017) found that work engagement mediated the relationship between psychological capital and organizational citizenship behavior. Sánchez-Cardona et al. (2023) confirmed that job resources are indirectly associated with the intention to stay in the organization through two mediators, meaningful work and work engagement.

Personal resources, as aspects of the self, refer to individuals’ sense of their ability to control and influence their environment (Hobfoll et al., 2003). They support goal achievement, protect against threats, reduce physiological and psychological costs, and promote professional growth and development (Xanthopoulou et al., 2007). Adaptable employees effectively manage change and uncertainty (Griffin et al., 2007). Job crafters proactively redesign their work, including tasks, workplace relationships, and cognitions about work, to increase meaning and compatibility (Wrzesniewski and Dutton, 2001). Employees who actively apply their professional strengths can achieve higher work outcomes and derive greater satisfaction from their work (Van Woerkom et al., 2016). In this study, we examine indirect associations between these work-related personal resources and perceived employability across the following aspects. Drawing on the Job Demands–Resources model, we expect that adaptability, job crafting, and strengths use may be positively associated with employee engagement at work. Work engagement activates professional skills and knowledge to meet work demands, stimulates the acquisition of new skills, and contributes to employee learning and professional growth. This helps implement work goals effectively and strengthens professional potential. In this way, personal resources may indirectly affect perceived employability through work engagement, which may serve as a mediating variable in the relationships among adaptability, job crafting, strengths use, and perceived employability.

*H5*: Work engagement mediates the relationship between employee adaptability and perceived external employability.

*H6*: Work engagement mediates the relationship between job crafting and perceived external employability.

*H7*: Work engagement mediates the relationship between employee strengths use at work and perceived external employability.

**Figure 1:**
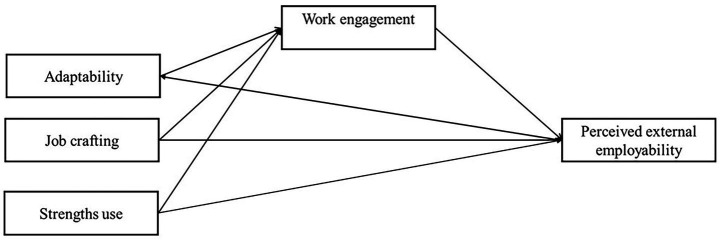
Research model.

The hypothesized research model is presented in Figure 1.

According to the research aim and hypotheses, the model suggests that adaptability, job crafting, and strength use behavior may predict perceived employability, and that work engagement may mediate the relationships between each personal resource and perceived employability.

## 3 Materials and methods

### 3.1 Research procedure and sample

This study was conducted in Lithuania from April to June 2025, and data were collected using a cross-sectional design by a market and public opinion research company. The survey was administered via Computer-Assisted Telephone Interviewing (CATI) and Computer-Assisted Web Interviewing (CAWI). The sample included 1,032 employees from state and private Lithuanian organizations. Our sample consisted of 492 (47.7%) men and 540 (52.3%) women, with ages ranging from 19 to 64 years (*M* = 42.5; *SD* = 11.85). 637 (61.7%) individuals had higher education, including university or non-university higher education, while 395 (38.3%) had vocational or secondary education. 374 (36.2%) worked in the public sector, 632 (61.2%) in the private sector, and 26 (2.5%) respondents indicated that they worked in the non-governmental sector. Regarding position, 266 (25.8%) of the study participants held managerial positions, while 766 (74.2%) held non-managerial positions. Length of organizational tenure ranged from 1 to 44 years (*M* = 9.54; SD = 8.94).

The research was conducted in accordance with the ICC/Esomar International Code on Market, Opinion and Social Research and Data Analytics (International Chamber of Commerce (ICC)/Esomar, 2025). Participants were informed that the study was conducted in accordance with research ethics requirements, that their participation was voluntary, and that they could withdraw from the study at any time. The anonymity and confidentiality of the study participants were ensured. Participants were informed that any personal information would not be disclosed when responding, and that the results would be analyzed in aggregate for scientific purposes only.

### 3.2 Measures

Respondents were asked to provide background information, including their gender, age, education, work sector, job tenure within the organization, and managerial position, and to respond to the questionnaire scales.

Adaptability, job crafting, strengths use, and perceived external employability were measured on a five-point Likert scale ranging from 1 (totally disagree) to 5 (totally agree). The response options for work engagement ranged from 1 (never) to 7 (always/every day). Cronbach’s alpha coefficients for all scales used in our study are shown in Table 1. The questionnaire was administered in Lithuanian, and all measures were translated from English into Lithuanian using a translation-back procedure.

**Table 1:** Means, standard deviations, and Pearson correlations between study variables (*N* = 1,032).

Variables	*M*	*SD*	1	2	3	4	5
Perceived employability	3.29	0.91	(0.929)				
Work engagement	4.45	1.09	0.407^**^	(0.878)			
Adaptability	3.65	0.69	0.424^**^	0.405^**^	(0.915)		
Job crafting	3.47	0.70	0.368^**^	0.443^**^.	0.464^**^	(0.901)	
Strengths use	3.62	0.71	0.366^**^	0.567^**^	0.460^**^	0.590^**^	(0.900)

Cronbach’s alpha coefficients are presented on the diagonal. **p < 0.01. M, mean; SD, standard deviation.

*Adaptability* was assessed using a five-item adaptability subscale (“I am well able to adjust to problems that confront me”) from the Adult Personal Resilience scale developed by Taormina (2015). The adaptability score was calculated as the average of responses to the scale items, with higher scores indicating stronger employee adaptability. The exploratory factor analysis confirmed a one-factor scale structure (Varimax rotation, KMO = 0.913, Bartlett’s test of sphericity Chi square = 4116.8, *df* = 15, *p* < 0.001) explaining 71.1% of variance with factor loadings ranging from 0.769 to 0.861.

*Job crafting* was measured using the four-item Broad Job Crafting Scale (BJCS) (“I make changes to my job to perform better”), developed by Vanbelle et al. (2017, 2023). The job crafting score was computed as the mean of responses to the scale items, with higher scores indicating stronger job crafting. The exploratory factor analysis confirmed a one-factor structure (Varimax rotation, KMO = 0.833; Bartlett’s test of sphericity, Chi-square = 2669.5, *df* = 6, *p* < 0.001), explaining 77.2% of the variance, with factor loadings ranging from 0.827 to 0.911.

*Strengths use* was assessed using the Strengths Use Behavior scale (“In my job, I make the most of my strong points”), developed by Van Woerkom et al. (2016). The original scale comprises six items; however, two items with the lowest factor loadings, as identified by the scale’s authors, were excluded. The scale score was computed by averaging responses, with higher scores indicating better application of an individual’s strengths at work. The exploratory factor analysis confirmed a one-factor structure (Varimax rotation, KMO = 0.834, Bartlett’s test of sphericity Chi-square = 2541.3, *df* = 6, *p* < 0.001), explaining 77.0% of the variance, with factor loadings ranging from 0.863 to 0.892.

*Work engagement* was assessed using the three-item Ultra-short Utrecht Work Engagement Scale (UWES-3) (Schaufeli et al., 2019), which measures the dimensions of energy (vigor), enthusiasm (dedication), and immersion (absorption) of engagement (“At my work, I feel bursting with energy”). An engagement score was calculated as the mean of responses to the three items, with higher scores indicating greater employee engagement at work. The exploratory factor analysis confirmed the scale’s one-factor structure (KMO = 0.717; Bartlett’s test of sphericity: Chi-square = 1698.0, *df* = 3, *p* < 0.001), accounting for 80.4% of the variance, with factor loadings ranging from 0.869 to 0.927.

*Perceived employability* was measured with a four-item Perceived external employability scale (“I could easily find another job, if I wanted to”) (De Cuyper et al., 2014; Vanhercke et al., 2014). The scale score was computed by averaging item responses, with higher scores indicating greater perceived employability. Exploratory factor analysis confirmed a one-factor structure for the scale (Varimax rotation, KMO = 0.862, Bartlett’s test of sphericity Chi square = 3288.7, *df* = 6, *p* < 0.001), explaining 82.5% of the variance, with factor loadings ranging from 0.896 to 0.918.

### 3.3 Data analysis

For this study, data analysis was conducted in three steps using IBM SPSS version 21 and IBM SPSS AMOS version 21. First, we examined the scales’ construct validity, reliability, and descriptive statistics (means, standard deviations, Pearson correlations, Student’s *t*-test). Reliability was assessed by calculating Cronbach’s alpha coefficients, and construct validity was evaluated using exploratory factor analysis. Hierarchical regression analysis was performed to identify predictors of work engagement and perceived external employability. To investigate predictors of work engagement, control variables were entered in the first step, followed by adaptability, job crafting, and strengths use behavior in the second step. To examine predictors of perceived external employability, it was regressed on the control variables (first step), followed by personal resources and work engagement (second step). Structural equation modeling (SEM) with 5,000 bootstrap resamples was conducted to test research hypotheses, primarily to evaluate the direct effects of employee adaptability, job crafting, and strengths use on perceived employability, and the mediating effect of work engagement in the relationships between personal resources and perceived employability. Age, gender, and education were included as covariates. Model fit was evaluated using multiple fit indices, including the chi-square statistic (*χ*^2^), the comparative fit index (CFI), the Tucker–Lewis index (TLI), and the root mean square error of approximation (RMSEA), with 90% confidence intervals. Bootstrapping with 5,000 resamples was used to generate bias-corrected 95% confidence intervals for indirect effects.

To mitigate the potential harmful effects of common method bias (CMB), respondents’ anonymity was maintained throughout data collection. Additionally, we conducted Harman’s single-factor test, as suggested by Podsakoff et al. (2024), to assess potential common method bias in the dataset. Items from all five scales were included in an unrotated exploratory factor analysis using principal axis factoring. The single factor explained 43% of the total variance. Since this is below the 50% threshold, the impact of common method bias is not considered a significant concern in this data (Podsakoff et al., 2024). We also conducted a single-factor CFA model in AMOS, with all items loading on a single latent factor, which showed poor fit (*χ*^2^/*df* = 44.10; CFI = 0.525; TLI = 0.469; RMSEA = 0.204). Conversely, a five-factor measurement model provided an acceptable fit (*χ*^2^/*df* = 1.84; CFI = 0.993; TLI = 0.990; RMSEA = 0.029). Variance inflation factors (VIFs), including those for the mediator work engagement, ranged from 1.367 to 1.867, all of which were below 5, indicating no multicollinearity issues. Cronbach’s alpha coefficients for all variables exceeded 0.88, reflecting good internal reliability of the scales.

## 4 Results

### 4.1 Descriptive statistics

Table 1 presents the means, standard deviations, and Pearson correlation coefficients between the study variables.

Intercorrelations indicate that all variables are positively and significantly associated. Correlations between perceived employability and other variables ranged from *r* = 0.366 (*p* < 0.01) for the use of the strengths to *r* = 0.424 (*p* < 0.01) for adaptability. Correlations between work engagement and personal resources ranged from *r* = 0.405 (*p* < 0.01) for adaptability to *r* = 0.567 (*p* < 0.01) for the use of strengths.

Comparing variables between the gender groups did not reveal any significant differences. Regarding age, negative correlations were found with employability (*r* = −0.239, *p* < 0.01) and job crafting (*r* = −0.069, *p* < 0.05), and a weak positive correlation with work engagement (*r* = 0.063, *p* < 0.05). As employees age, their likelihood of being hired by other employers decreases, and they become less involved in job crafting, yet their overall engagement with work slightly increases, although not all of these connections were strong. Regarding education, it was found that education was positively, but not strongly, correlated with job crafting (*r* = 0.104, *p* < 0.01) and strengths use (*r* = 0.104, *p* < 0.01).

Summarizing the relationships between demographic characteristics and the independent variables analyzed in this study, it is essential to note that the observed significant correlations were generally weak, and there were no significant differences in mean values across gender groups. Regarding the dependent variable, perceived employability, younger employees reported higher evaluations of their personal ability to be employed by other employers, whereas gender and education were not significantly associated with the employability assessment. Perceived employability was rated slightly higher by respondents working in the private sector than by those in the public sector organizations (*M* = 3.34 and *M* = 3.21, respectively; *p* = 0.034), although the difference is small. In this study, the private sector sample is nearly twice as large as the public sector sample; therefore, the results are insufficient to determine whether the organizational sector is significant for employees’ perceived employability. Further research are necessary to examine these relationships in more detail.

### 4.2 Predictors of work engagement and perceived employability

At the next stage of analysis, we tested four hierarchical regression models to examine predictors of work engagement and perceived employability, with gender, age, and education included as control variables. For work engagement as the dependent variable, control variables were entered in Step 1, and the main study variables were added as independent variables in Step 2. In Step 3, perceived employability was regressed on the control variables, and in Step 4, to predict employability, all main study variables were added. The results are presented in Table 2.

**Table 2:** Hierarchical regression models testing predictors of employee work engagement and perceived employability.

Control and independent variables	Dependent variables			
	Work engagement		Perceived employability	
	Step 1	Step 2	Step 3	Step 4
	*β*	*β*	*β*	*β*
Gender	−0.009	−0.006	0.048	0.042
Age	0.058	0.053	−0.233***	−0.238***
Education	−0.056	0.002	−0.021	−0.016
Adaptability		0.155***		0.240***
Job crafting		0.128***		0.094**
Strengths use		0.418***		0.077*
Work engagement				0.241***
*R* ^2^	0.007	0.359	0.056	0.320
Δ *R*^2^		0.352		0.263
*F*	(3, 1,028) 2.443, *p* = 0.063	(6, 1,025) 95.759***	(3, 1,028) 20.412***	(7, 1,024) 68.730***
Cohen’s *f*^2^	0.007	0.560	0.059	0.471

*p < 0.05; **p < 0.01; ***p < 0.001; *β*, standardized beta coefficient; VIF (Variance Inflation Factor) coefficients for the independent variables in every model did not exceed the statistical level of 2.0.

As shown in Steps 1 and 2 (Table 2), after controlling for demographic variables, adaptability, job crafting, and strengths use positively affected employee work engagement, accounting for 35.9% of its variance. Data in Steps 3 and 4 showed that adaptability, job crafting, strengths use, and engagement positively affected perceived employability, explaining 32.0% of its variance. Notably, the direct relationship between strengths use and employability was weak (*β* = 0.077, *p* < 0.05), the weakest of the three compared with adaptability and job crafting. The effect sizes for gender, age, and education on the dependent variables in Steps 1 and 3 were small (Cohen’s *f^2^* ranged from 0.007 to 0.059). After adding independent predictors in Steps 2 and 4, the effect size increased to large when predicting engagement (Cohen’s *f^2^* = 0.560) and employability (Cohen’s *f^2^* = 0.471). The regression results allow us to draw preliminary conclusions regarding hypotheses H1–H3, suggesting that personal resources (adaptability, job crafting, and strengths use) are positively associated with perceived employability. Additionally, preliminary findings suggest a positive relationship between engagement and employability. These relationships were examined in more detail using structural equation modeling.

Regarding the research hypotheses, we tested a model with both direct and indirect relationships among employee adaptability, job crafting, and strengths use as independent variables, perceived employability as the dependent variable, and work engagement as a mediator (see Figure 1). The initial hypothesized model, which included direct paths from adaptability, job crafting, and strengths use to employability, and indirect paths through work engagement, did not fit the data well, *χ*^2^ (0) = 0.0, CFI = 1.00, TLI = 0.00, RMSEA = 0.384. The direct path from strengths use to perceived employability was very weak (*β* = 0.076, *p* = 0.034); therefore, it was removed, resulting in a final, more parsimonious model with good overall fit, *χ*^2^ (1) = 1.92, *p* = 0.166, CFI = 0.999, TLI = 0.994, RMSEA = 0.030, CI [0.00; 0.094]. Conceptually, this modification is consistent with theoretical assumptions that the relationship between the use of strengths and employability is fully mediated by work engagement, and that the use of personal strengths at work enhances employability primarily through work engagement, rather than directly. That is, using one’s strengths at work predicts motivational and affective states (e.g., immersion, energy, enthusiasm), which, in turn, predict higher perceptions of employability, indicating a fully mediated relationship.

Table 3 presents the standardized weight estimates from the AMOS mediation regression, along with their confidence intervals, which describe the total, direct, and indirect effects of adaptability, job crafting, strengths use, and engagement on perceived employability.

**Table 3:** Direct, indirect, and total effects of adaptability, job crafting, strengths use, and work engagement in predicting perceived employability.

Independent variables	Mediator	Dependent variable	Direct effect	Indirect effect	Total effect
			*β*, 95% CI	*β*, 95% CI	*β*, 95% CI
Adaptability	Work engagement	Perceived external employability	0.261*** [0.193, 0.330]	0.037*** [0.020, 0.058]	0.298*** [0.232, 0.364]
Job crafting			0.141*** [0.064, 0.215]	0.029*** [0.014, 0.050]	0.170*** [0.095, 0.244]
Strengths use			0.000	0.101*** [0.068, 0.145]	0.101*** [0.068, 0.145]

***p < 0.001, *β*, standardized beta coefficients, 95% CI, confidence intervals.

**Figure 2:**
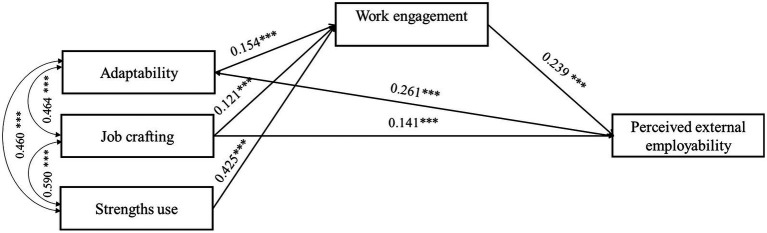
Empirical structural model presenting direct and indirect standardized effects of adaptability, job crafting, and strengths use on perceived external employability. ***p < 0.001.

The final research results are presented in Figure 2.

The results indicate that adaptability and job crafting have direct positive effects on perceived employability, supporting hypotheses H1 and H2. Strengths-based behavior did not directly predict employability; thus, H3 was not confirmed. Personal resources, including adaptability, job crafting, and the use of strengths at work, were positively related to work engagement (*β* = 0.154, *p* < 0.001; *β* = 0.121, *p* < 0.001; and *β* = 0.425, *p* < 0.001, respectively). The fourth hypothesis (H4), which posits a positive relationship between work engagement and perceived employability, was also confirmed: the standardized effect of work engagement on employability was positive and significant. (*β* = 0.239, *p* < 0.001). Employees who successfully adapt to the demands of work and constant change (adaptability), proactively align their capabilities with work tasks and modify work processes to achieve better results (job crafting), and reasonably assess their personal strengths, identifying and utilizing them in their work (strengths use), are more motivated, energetic, and dedicated. Moreover, engagement, which describes the energizing, positive motivational potential of an employee, is positively related to confidence in personal employment opportunities in the external labor market.

As shown in Table 3, the indirect effect of employee adaptability on perceived employability through work engagement was significant. (*β* = 0.037, *p* < 0.001, CI [0.020, 0.058]). Although the total effect of adaptability on employability was significant (*β* = 0.298, *p* < 0.001), the direct effect was also significant yet smaller (*β* = 0.261, *p* < 0.001). Therefore, work engagement partially mediated the relationship between adaptability and perceived external employability, supporting H5. Another significant mediating effect of engagement was observed in the relationship between job crafting and employability (*β* = 0.029, *p* < 0.001, CI [0.014, 0.050]). The total effect of job crafting on employability was significant (*β* = 0.170, *p* < 0.001). After including the mediator, the effect decreased, but remained significant (*β* = 0.141, *p* < 0.001). Thus, engagement partially mediated the relationship between job crafting and employability, supporting H6.

Finally, consistent with H7, the mediation analysis showed that the relationship between strengths use behavior and employability was mediated by engagement (*β* = 0.101, *p* < 0.001, CI [0.068, 0.145]). The total effect was significant (*β* = 0.101, *p* < 0.001); however, the direct effect of strengths use on employability was not confirmed. Thus, engagement fully mediated the relationship between the use of employee strengths and perceived external employability, supporting H7.

## 5 Discussion

In today’s dynamic labor market, employees’ perceptions of their external employability - the belief in the ability to find employment outside the current organization - are increasingly important (Fugate et al., 2004; Vanhercke et al., 2014). The present study was conducted with a sample of Lithuanian employees and focuses on the relationships between perceived external employability and work-related personal resources. Drawing on the Job Demands—Resources model (Bakker and Demerouti, 2017; Bakker et al., 2023), we proposed that personal resources, such as adaptability, job crafting, and strengths use at work, relate to perceived external employability directly and indirectly through work engagement, an active motivational state that may explain the underlying mechanism of those relationships. More specifically, adaptability, job crafting, strengths use, and work engagement were analyzed as predictors of perceived external employability. Furthermore, work engagement was analyzed as an intervening variable (mediator) in the relationships of adaptability, job crafting, and strengths use with external employability.

The first hypothesis (H1) examines the direct positive relationship between adaptability, defined as an individual’s ability to effectively adapt to job demands, roles, and environments (Griffin et al., 2007), and perceived employability. The study found that adaptability is positively related to perceived employability, thereby confirming the first hypothesis. This result is consistent with findings from other authors (Forrier et al., 2009, 2015). Adaptability, as the active acceptance of changes at work, the adjustment of attitudes and behaviors, the acquisition of new knowledge, or the learning of new skills, helps employees accumulate useful experience, expand the range of professional competencies, and serve as an internal force that reveals professional opportunities in the external labor market (Collie et al., 2020; Pulakos et al., 2000).

The direct positive relationship between job crafting and perceived employability supports the study’s second hypothesis (H2). Employees who proactively shape their work, modify job requirements and resources (Tims and Bakker, 2010), and improve work design and the social environment (Wrzesniewski and Dutton, 2001) report higher perceived employability in other organizations. This result aligns with previous studies. For example, Sartori et al. (2023) examined how job-crafting training affects its applicability and, in turn, self-perceived employability. The authors concluded that by learning and performing more complex tasks, employees build a professional foundation necessary for sustainable employability or promotion. The positive effect of job crafting on employability was also confirmed in Signore et al.'s (2023) study. Akkermans and Tims (2017) operationalized subjective career success as perceived employability and found that career competencies lead to higher employability evaluations through job crafting, which serves as a mediator in this relationship. Irfan and Qadeer (2020) also found that employee involvement in job crafting behaviors led to sustainable employability during the COVID-19 pandemic. If adaptability refers to one’s ability to adapt to work, then job crafting is about a person’s ability to adapt a job to themselves. Both processes help test and expand the boundaries of professional opportunities and, at the same time, lead to a stronger confidence in individuals’ ability to be employed by multiple organizations.

The third hypothesis (H3) examined the relationship between using strengths at work and perceived employability. As noted in the theoretical section, personal resources are aspects of the self that reflect individuals’ sense of their ability to control and influence their environment successfully (Hobfoll et al., 2003). According to the Performance theory (Griffin et al., 2007), work processes are characterized by stability and uniformity on the one hand and by the constant likelihood of environmental and functional changes on the other. Thus, professional strengths are inner forces that help individuals adjust to, incorporate into, and perform effectively within these processes. However, research on the relationship between strengths use and perceived employability is among the least studied, compared with adaptability or job crafting. After reviewing the scientific literature, we identified only a few studies that examined these relationships and reported a positive association between strengths use and perceived employability (Gürbüz et al., 2022b; Matsuo, 2022). In our study, a positive linear relationship was first observed between strengths use and employability (*r* = 0.366, *p* < 0.01; see Table 1). However, when testing the hypothesized research model (see Figure 1), which included all three independent variables and work engagement as a mediating variable, the linear relationship between strengths use and perceived employability was not confirmed (see Table 3 and Figure 2). Thus, we conclude that the third research hypothesis (H3) regarding the relationship between strengths use and employability was not supported. This result suggests a more complex relationship among these constructs, so we tested this by treating work engagement as an intermediate variable.

The fourth hypothesis (H4) was also confirmed, stating that work engagement is positively correlated with perceived employability. Employees who find their work stimulating, feel vigorous and energetic, and enjoy working have a higher view of their personal employability in the external labor market. This result aligns with findings from other studies. For example, Rahi (2022) found that work engagement positively impacts sustainable employability. It is also worth noting that the relationship between work engagement and employability can run in the opposite direction, with employability predicting work engagement (Gürbüz et al., 2023). In other studies, employability was found to moderate the relationships between employee health and workplace relational civility and the dependent variable, work engagement (Liu et al., 2022; Zhang and Liu, 2023). Overall, the diversity of relationships between work engagement and perceived employability, as revealed across various studies, supports the need for further research.

To evaluate the mediating role of work engagement in predicting perceived employability, three hypotheses were proposed: that work engagement mediates the relationships between adaptability, job crafting, and strengths use and employability. The results confirm that engagement partially mediated the relationships between adaptability and employability (H5) and between job crafting and employability (H6). Regarding the seventh hypothesis, the study found that engagement fully mediated the relationship between strengths use and employability (see Table 3), confirming H7. This suggests that using one’s strengths does not directly predict perceptions of employability but operates primarily through increased engagement. Adaptability, job crafting, and strengths use were indirectly related to higher perceived external employability through stronger work engagement, although the direct effects of adaptability and job crafting on perceived employability also remained significant.

The current findings should also be considered within the context of Lithuania’s labor market. Lithuania is a relatively small and open economy that has experienced significant labor market changes over the past decades. The transition from a centrally planned to a market economy has been accompanied by increased labor mobility, emigration flows, demographic aging, skills mismatches, and labor shortage in several sectors (Labour Market in Lithuania, 2023; OECD, 2025). Such conditions may increase the importance of individual career self-management and personal resources in maintaining employability in a dynamic labor market. Within this context, proactive behavior at work, which includes employees’ ability to adapt to a dynamic work environment and requirements, job improvements through job crafting, and the utilization of strengths, not only enhances their motivational state—work engagement, but also helps to understand and assess their personal professional potential and may increase confidence in being capable to obtain and maintain employment in the external labor market.

At the same time, the theoretical mechanism explored in this research is based on a widely accepted framework, such as the Job Demands–Resources model (Bakker and Demerouti, 2017). This perspective suggests that personal resources can foster work engagement, which, in turn, supports positive career-related outcomes, including perceptions of employability. Therefore, similar relationships can also be expected in other studies conducted with employee samples from organizations operating in different labor markets. However, the specific outcomes may vary depending on the institutional conditions, labor market regulations, or cultural features. Future research could explore whether similar work-related personal antecedents of perceived employability exist across different national contexts to further examine potential cross-country differences.

In summary, our study’s results indicate that personal resources, such as adaptability, job crafting, and the use of strengths, predict positive perceptions of employability, both directly and indirectly by strengthening work engagement, a motivational state that mediates these relationships. Employees who are open to changes in work and the environment, proactive when it comes to new, unfamiliar tasks, and who initiate physical and cognitive changes in their tasks, consider and apply personal strengths in the performance process, are more engaged in their work—more vigorous, energized, and absorbed, and have a positive attitude toward their work. In turn, engaged employees have a more positive view of their personal employment opportunities in the external labor market and may be more self-confident in their ability to successfully compete for jobs in other organizations.

## 6 Limitations and suggestions for future studies

When presenting the results of this study, it is important to discuss the limitations and directions for further research. We found that perceived employability did not differ by age, gender, education, or place of residence; however, the public or private sector in which the organization operates, professional characteristics, such as the person’s profession or position held, may also be significant and can be analyzed in more detail in further studies. In terms of employment status, our study focused on employed individuals; therefore, broader studies of the assumptions underlying perceived employability in samples of students or graduates, as well as the unemployed, are relevant. This would help reveal the factors underlying the strengthening of employability across different employment status groups, its impact on career decision-making and active job search, and, more broadly, its impact on the supply of specialists of professions in demand in the labor market.

This study used a cross-sectional design, in which data were collected at a single point in time. Longitudinal research on perceived employability assumptions is also relevant, as it reveals the dynamics of these phenomena over time, their causal relationships, and is relevant in at least three aspects. First of all, the perceived employability assumptions examined in this study (job crafting, strengths use behaviors, and even adaptability), as well as employability perceptions, are not fixed phenomena and can change over time. This justifies the relevance of longitudinal research, especially when linking improvements in employability to organizational- and individual-level interventions aimed at developing personal competencies, shaping working conditions, and addressing other factors. Secondly, longitudinal studies provide an opportunity to supplement subjective assessments of employability with objective indicators of changes in employment status among employed, graduates entering the labor market, and unemployed individuals over a given period. The third aspect concerns perceived employability outcomes, including work performance, engagement, professional resilience, job insecurity, or intention to leave the organization. Longitudinal studies of these relationships would confirm the significance of perceived employability for employees and the organization, and the measures that strengthen it in the long term.

The employees who participated in the study were the only source of information that revealed subjective perceptions of personal experiences. Interpretation of the findings may be limited when results are based solely on the participants’ self-observation. The impact of this limitation on the results may be mitigated by using additional sources of information. These may include objective indicators of work performance, career advancement, or other professional achievements. Combining quantitative and qualitative research methods can also provide additional insights into employees’ experiences and their significance for perceived employability.

Future research should also focus on a broader range of factors that enhance or limit perceived employability, as well as on mediators and contextual conditions. From both theoretical and practical perspectives, research examining combinations of organizational, leadership, work, and individual factors, in which they may supplement each other or, conversely, may act as restraining forces that limit each other’s positive effects on employability, is also valuable.

## 7 Implications

From a theoretical perspective, the focus on work-related personal resources and work engagement confirms the value of applying the Job Demands-Resources model to investigate the antecedents of perceived employability (van Hetty Emmerik et al., 2012) and complements previous research in this field in several ways.

First, the received results add to studies on the personal predictors of perceived employability, as the group of personal resources in the Job Demands—Resources model is complemented by characteristics such as adaptability, job crafting, and strengths use behaviors.

Second, personal resources are traditionally conceptualized as antecedents of work engagement and performance-related outcomes (Xanthopoulou et al., 2007). Our study demonstrates that adaptability, job crafting, and strengths use predict employees’ perceptions of employability in the external labour market and, in this way, expand the outcome domain of the Job Demands -Resources model by including career-related outcomes. This suggests that the motivational pathway strengthens in-role functioning, well-being, and positive attitudes towards work and the organization (Bakker et al., 2023); however, it is not limited to the work process and may also impact employees’ broader perceptions of their labor market value.

Third, we found that personal resources are not only directly but also indirectly related to perceptions of employability through the mediator work engagement. These resources primarily function as predictors of the motivational states that energize employees and promote engagement. Engaged employees are more likely to experience positive emotions, demonstrate initiative, and feel capable of adapting to changing work demands, which, in turn, enhances their perceptions of employability. Therefore, the analyzed resources appear to contribute to employability indirectly by nurturing motivational and affective processes that make individuals more proactive and confident at work and also in managing their careers. This shows that personal resources in the Job Demands-Resources model operate through multiple pathways, complementing the motivational process, and reveals that engagement is the main, but not the only, mechanism linking personal resources with career-related outcomes.

Finally, it is important to note that this study is one of the first conducted in Lithuania to examine employees’ perceived external employability and its personal prerequisites. This opens up opportunities for future research to clarify the antecedents and directions for enhancing the perceived employability of labor market participants across various employment statuses. Adaptability, crafting, and using strengths are not limited to work activities; a person can apply these qualities in various contexts, for example, in studies or a job search. Thus, the principles of the Job Demands-Resources model developed for the work environment can also be applied to other activities related to job search or employment. Strengthening the perceived employability resources analyzed in this study can be useful not only for those already working but also for graduates starting their professional careers or for unemployed persons engaged in job search activities.

In conclusion, our study complements existing research in this field and highlights the importance of personal facets associated with work, thereby strengthening the potential for employability in the external labor market. Research results contribute to understanding whether adaptability, job crafting, and strengths use, as work-related personal resources, together with work engagement, which reflects the motivational path in the Job Demands-Resources model, may be considered when analyzing individual-level predictors of employee perceived external employability in the labor market.

The research findings offer several practical implications for organizations, HR professionals, managers, as well as employees seeking to increase employee engagement and long-term career sustainability. By showing that adaptability, job skill development, and leveraging strengths positively predict employees’ perceived employability—both directly and through job engagement—the study underscores the strategic value of investing in personal resource development.

Organizations should view employees’ personal resources as strategic capabilities that can be developed. Training interventions can be designed to enhance adaptability through change-readiness and resilience-improving workshops, rotational assignments that expose employees to diverse tasks and contexts, to build job-crafting skills by teaching employees to proactively adjust task boundaries, manage relationship interactions, to promote strengths use through strengths assessments, coaching conversations, and job redesign initiatives that align roles with employees’ core competencies. From a Job Demands—Resources perspective (Bakker et al., 2023), strengthening these personal resources enhances the motivational process that drives engagement. From a Conservation of Resources perspective (Hobfoll et al., 2018), such investments help employees build resource reserves that generate further benefits over time, including stronger perceptions of employability.

The role of line managers is particularly important. Leaders can strengthen adaptability, job crafting, and strengths use by encouraging employees to actively participate in implementing changes, discussing current work challenges and obstacles with them, providing support when needed to solve complex work issues, and promoting their innovative behavior and professional growth. The autonomy and feedback provided at work help employees adapt their work flexibly, enabling them to better apply their knowledge and skills, achieve goals effectively, and meet their most important personal needs. Strengths-based leadership motivates employees to engage in tasks aligned with their strengths, fosters a positive psychological climate within the work group, and encourages colleagues to complement one another’s professional strengths in joint projects. This helps reinforce an attitude toward developing professional strengths as an organizational value, since, from a positive psychology perspective, it is easier to improve than to work on weaknesses (Bakker and van Woerkom, 2018; Ghielen et al., 2018).

Employees must also take responsibility for their employment security, as no organization can guarantee a job for life. The changing nature of work, rapid technological innovations, and shifts in the business environment affect the labor market, job demand, and the professional training requirements for job seekers. According to Van Dam (2004), organizational employability should be distinguished from individual employability, meaning that employees themselves must be actively involved in managing their careers and, first of all, in self-development and continuous learning. In this way, the conditions that support employability combine organizational measures that promote professional development with employees’ participation in activities that strengthen their professional potential.

## 8 Conclusion

A study of Lithuanian employees found that work–related personal resources - adaptability, job crafting, and strengths use - were positively associated with work engagement and perceived external employability. Work engagement partially mediated the relationships of adaptability and job crafting with employability, and fully mediated the link between strengths use and employability, highlighting the motivational pathway proposed by the Job Demands–Resources framework. Engaged employees are more likely to experience positive emotions, demonstrate initiative, and feel capable of adapting to changing work demands. Adaptability, job crafting, and strengths use contribute to work engagement by nurturing motivational and affective processes that, in turn, strengthen perceived employability and make employees more proactive and confident in managing professional careers. These results extend the theory by showing that self-perceptions of one’s employment prospects are a key outcome of employees’ resource investment and engagement. Practically, the findings suggest that organizations and employees can enhance perceived employability by developing a range of personal resources, such as adaptability, job crafting, and strengths use, and by promoting work engagement, a link that connects these resources to perceived employability.

## Data Availability

The raw data supporting the conclusions of this article will be made available by the authors, without undue reservation.
